# Increasing Patient Safety and Treatment Quality by Using Intraoperative MRI for Organ-Preserving Tumor Resection and High-Dose Rate Brachytherapy in Children with Bladder/Prostate and Perianal Rhabdomyosarcoma

**DOI:** 10.3390/cancers15133505

**Published:** 2023-07-05

**Authors:** Andreas Schmidt, Constantin Roder, Franziska Eckert, David Baumann, Maximilian Niyazi, Frank Fideler, Ulrike Ernemann, Marcos Tatagiba, Jürgen Schäfer, Cristian Urla, Simon Scherer, Jörg Fuchs, Frank Paulsen, Benjamin Bender

**Affiliations:** 1 Department of Pediatric Surgery and Pediatric Urology, University Children’s Hospital, Eberhard Karls University Tuebingen, 72076 Tuebingen, Germany; 2 Department of Neurosurgery, University Hospital, Eberhard Karls University Tuebingen, 72076 Tuebingen, Germany; constantin.roder@med.uni-tuebingen.de (C.R.);; 3 Center for Neuro-Oncology, Comprehensive Cancer Center Tuebingen-Stuttgart, University Hospital, Eberhard Karls University Tuebingen, 72070 Tuebingen, Germanybenjamin.bender@med.uni-tuebingen.de (B.B.); 4 Department of Radiation Oncology, University Hospital, Eberhard Karls University Tuebingen, 72076 Tuebingen, Germanyfrank.paulsen@med.uni-tuebingen.de (F.P.); 5 Department of Radiation Oncology, AKH, Comprehensive Cancer Center Vienna, Medical University Vienna, 1090 Vienna, Austria; 6 Department of Anesthesiology and Intensive Care Medicine, University Hospital, Eberhard Karls University Tuebingen, 72076 Tuebingen, Germany; 7 Department of Diagnostic and Interventional Neuroradiology, University Hospital, Eberhard Karls University Tuebingen, 72076 Tuebingen, Germany; 8 Department of Pediatric Radiology, University Hospital, Eberhard Karls University Tuebingen, 72076 Tuebingen, Germany; 9 Center for Pediatric Oncology, Comprehensive Cancer Center Tuebingen-Stuttgart, University Hospital, Eberhard Karls University Tuebingen, 72070 Tuebingen, Germany

**Keywords:** rhabdomyosarcoma, bladder/prostate, perianal, brachytherapy, intraoperative MRI

## Abstract

**Simple Summary:**

The combination treatment of organ-preserving tumor resection and brachytherapy in children with bladder/prostate and perianal rhabdomyosarcoma can reduce therapy-associated side effects while maintaining excellent oncological outcome. This highly individualized hybrid treatment concept poses specific challenges for all clinicians involved in the local treatment. The aim of this study was to determine whether the use of an intraoperative MRI can improve the clinical workflow. These findings may have a positive impact on the treatment quality and patient safety of children with bladder/prostate and perianal RMS.

**Abstract:**

In children with bladder/prostate (BP) and perianal rhabdomyosarcoma (RMS), we use a hybrid treatment concept for those suitable, combining organ-preserving tumor resection and high-dose rate brachytherapy (HDR-BT). This treatment concept has been shown to improve outcomes. However, it is associated with specific challenges for the clinicians. The exact position of the tubes for BT is a prerequisite for precise radiotherapy. It can finally be determined only with an MRI or CT scan. We evaluated the use of an intraoperative MRI (iMRI) to control the position of the BT tubes and for radiotherapy planning in all patients with BP and perianal RMS who received the above-mentioned combination therapy in our department since January 2021. iMRI was used in 12 children. All tubes were clearly localized. No adverse events occurred. In all 12 children, radiotherapy could be started on time. In a historical cohort without iMRI, this was not possible in 3 out of 20 children. The use of iMRI in children with BP and perianal RMS improved patient safety and treatment quality. This technology has proven to be successful for the patient population we have defined and has become a standard procedure in our institution.

## 1. Introduction

Rhabdomyosarcoma (RMS) is the most common soft tissue sarcoma in children [1]. Multimodal treatment concepts consist of chemotherapy followed by local therapy comprising surgery and/or radiotherapy [2,3]. International and interdisciplinary efforts have resulted in an overall 5-year survival rate higher than 70% [1]. Among the disciplines involved in the treatment, a focus has been put on the prevention of therapy-associated complications [4,5,6], including organ preservation and the reduction of radiotherapy-associated sequelae.

The combination treatment of organ-preserving tumor resection and brachytherapy (BT) has been established for selected tumor sites when certain criteria are met [7,8,9]. For RMS in the area of the urinary bladder/prostate (BP), this procedure can be considered a standard procedure nowadays [10,11]. In perianal RMS, which is less frequent but has a considerably worse prognosis, this kind of treatment is also used in individual cases [8,9,12,13]. BT offers advantages compared to conventional radiation modalities in terms of focusing the radiation on the target volume and sparing surrounding healthy structures [14,15]. 

Especially for BP-RMS, two different BT treatment combinations have been published in larger numbers of cases, which vary predominantly with regard to the extent of surgery and the irradiation technique used [8,16]. In our department, we aim for a marginal R0 resection and use individually 3-dimensional (3D)-planned high-dose rate (HDR)-BT. A major challenge, besides the actual organ-preserving tumor resection and the subsequent reconstruction of the organ by the surgeon, is the exact placement of the BT tubes around the former tumor area, without injury to the adjacent healthy organs, in typically young children with correspondingly small anatomical conditions. Incorrect positioning of the tubes would lead to inadequate irradiation of the target volume as well as unnecessary irradiation of the surrounding healthy structures and therefore must be avoided. The exact position of the tubes can only be determined with certainty by means of an MRI or CT scan. Up until now, we have carried out an MRI outside the operation room (OR) unit. If a tube revision was necessary, the patient had to be brought back to the OR. In order to improve the patient safety and the treatment quality, we evaluated the use of intraoperative magnetic resonance imaging (iMRI) for controlling the position of the surgically inserted BT tubes and planning the irradiation. To our knowledge, this is the first report on the use of iMRI in children with BP and perianal RMS. 

iMRI was initially developed for neurosurgical procedures and is frequently used in combination with neuronavigation. Indications for its use in children are gliomas, pituitary adenoma, vascular diseases, and epilepsy surgery [17,18,19]. In most cases, it serves to control the extent of resection and correct anatomical changes after tumor resection due to the so-called brain shift [20,21]. 

## 2. Materials and Methods

All patients with BP and perianal RMS who qualified for organ-preserving tumor resection and BT, and were treated in our department since January 2021 were prospectively registered in this study. A historical cohort consisted of all patients who received analogous treatment from 2009 to December 2020, in which the MRI for controlling the position of the BT tubes was performed outside the OR unit [10]. Patients who received only a CT scan to control the BT tube position and for BT planning are not included in the study (10 BP-RMS, 3 perianal RMS). The data analysis was done retrospectively. The study was approved by the institutional ethics committee (No. 293/2023BO2). 

All patients received risk-adjusted neoadjuvant chemotherapy according to the current Cooperative Weichteilsarkom Studiengruppe (CWS) protocol [22]. 

The local therapy was determined in a multidisciplinary tumor board (MDT). It was primarily based on the preoperatively evaluated tumor extension and the intraoperative findings. Patients were included if organ-preserving tumor resection with subsequent HDR-BT was performed.

**Figure 1:**
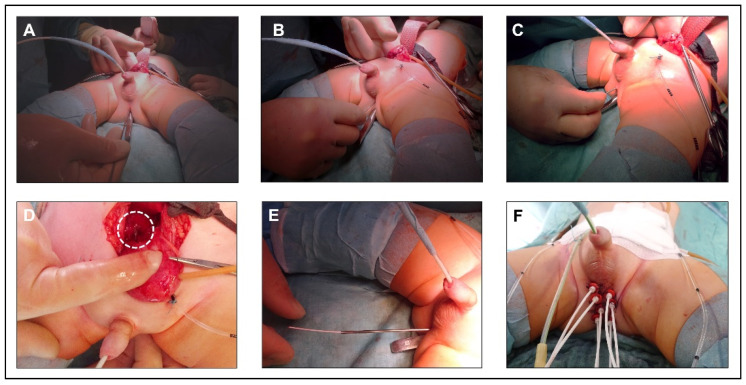
Principle of brachytherapy tube placement. (**A**–**C**) insertion of a sharp cannula into the small pelvis through the perineum. For identification, a metal probe was inserted into the rectum. (**D**) tip of the cannula (dashed line) in the small pelvis behind the urinary bladder. (**E**) insertion of the BT tube through the cannula. (**F**) perineum after placement of 7 BT tubes and fixation to the skin. An additional tube was inserted transurethrally.

For irradiation in BP-RMS patients, BT tubes are placed after tumor resection around the former tumor area, with an additional tube being inserted transurethrally in individual cases (Figure 1). The tube placement is performed in close cooperation and coordination between the surgeon and the treating radiation oncologists. A sharp cannula is inserted into the small pelvis through the perineum (Figure 1A–D). The BT tube is then placed through this cannula and fixed to the outside of the skin as well as inside the body with absorbable sutures (Figure 1E,F). Children under 3 years of age pose a particular challenge due to the anatomic conditions of a small caliber urethra and a narrow retrourethral spatium, with an increased risk of perforation of the rectum and injury to the corpora cavernosa. After tumor resection in children with perianal RMS, we place the tubes at the resection margin and fix them there. With the individually 3D-planned HDR-BT technique used, the distance between the tubes should not exceed 1 cm, as otherwise an excessively high punctual irradiation dose is necessary to adequately cover the previously determined target volume. BT should typically start on the second day after surgery. In fractions of 3 Gy, administered twice daily, the children received a total dose of 36 Gy. Details of the protocol have been described before [8].

To control the position of the BT tubes and to plan the irradiation, an MRI and a CT scan are performed after the placement of the tubes. The additional CT scan is necessary for the exact contouring of the tubes and the BT planning as well as radiation dose calculation. 

**Figure 2:**
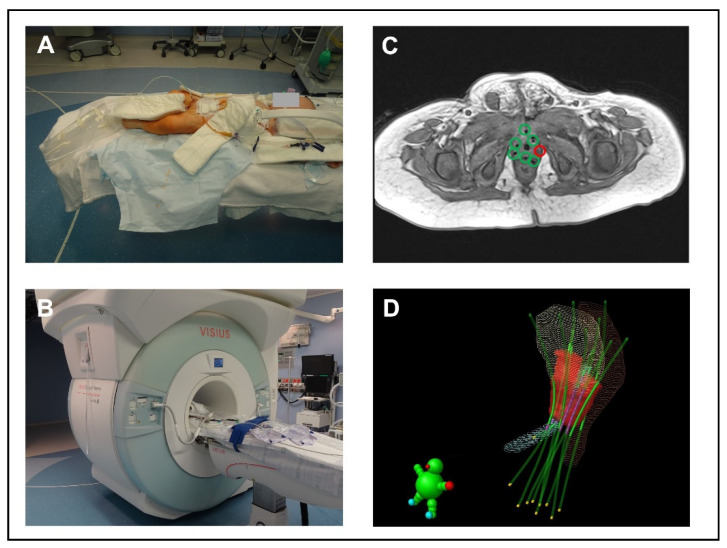
Principle of intraoperative MRI. (**A**) iMRI suite; position of a child on the table extension designed for this specific purpose. (**B**) “scanning position” of the magnet. (**C**) iMR image with BT tubes. The green marked tubes are in the correct position; a tube is missing at the red marked site. (**D**) 3D image of the BT tube location in relation to the surrounding anatomical structures in the radiotherapy planning system. Red volume = target volume, blue volume = urethra, yellow volume = urinary bladder, brown volume = rectum, green lines = BT tubes.

A modified ceiling-mounted, moveable 1.5 T magnet (Espree; Siemens Medical Systems, Erlangen, Germany) was used for iMRI (Figure 2). It is located in an intraoperative MR suite (IMRIS Visius Surgical Theatre; IMRIS Inc., Winnipeg, MB, Canada) [23]. The magnet is located in a “parking bay” with shielded doors. In this condition and with the doors closed, the adjacent OR can be used in a regular manner. Once the magnet moves to its “scanning position”, all ferromagnetic equipment must be placed outside the 5 Gauss line. For neurosurgical procedures, the patient’s head is placed at the top of the table in an MR-compatible DORO skull clamp with disposable skull pins (ProMed Instruments GmbH, Freiburg, Germany). Since the “scanning position” of the magnet is predetermined and cannot be changed, we placed the children in our study the other way around on the operating table, meaning with their feet pointing towards the MRI (Figure 2A,B). To prevent the feet from hanging in the air, a non-ferromagnetic, MR-compatible table extension was designed specifically for this purpose. All monitoring equipment was MR-compatible, and hearing protection was achieved by using ear plugs (Ohropax Yellow, OHROPAX GmbH, Wehrheim, Germany) combined with neonatal noise attenuators (MiniMuffs, Natus Medical Incorporated, Oakville, ON, Canada). Adequate preparation of the patient and the OR was checked with standardized MR safety checklists, before moving the patient into the scanning position. 

The MRI protocol included, in all patients, a transversal T2-weighted turbospin-echo (TSE) sequence (49 slices, 3 mm slice thickness, repetition time (TR) 10,860 ms, echo time (TE) 127 ms, field-of-view (FoV) 180 × 153 mm^2^, in-plane resolution 0.56 × 0.56 mm^2^), a sagittal T2-weighted TSE (29 slices, 3 mm slice thickness, TR 5500 ms, TE 129 ms, FoV 180 × 151 mm^2^, in-plane resolution 0.47 × 0.47 mm^2^), and a T1-weighted transversal TSE sequence (49 slices, 3 mm slice thickness, TR 661 ms, TE 15 ms, FoV 160 × 148 mm, in-plane resolution 0.5 × 0.5 mm^2^) that cover the complete pelvic region. Depending on preoperative imaging, additional diffusion-weighted images, T1 fat saturated images or T2-weighted fat saturated images, were acquired on a case-by-case basis.

## 3. Results

### 3.1. Patients

Over a period of 25 months, 12 children (12 boys) were included in this study. Median age at time of surgery and BT was 28 months (range 13–73). Ten of the patients had a BP-RMS and two patients had a perianal RMS. All tumors were of the embryonal subtype. After a median follow-up of 10.5 months (range 6–23), 10 patients are still alive and show no evidence of tumor relapse (Table 1). One of the patients with BP-RMS experienced an early relapse and died despite maximum therapy due to tumor progress. A second patient experienced an intestinal obstruction with a septic shock seven months after tumor resection and died. One patient with BP-RMS developed urethral stenosis in the postoperative course. He has since been successfully operated on and can void without any problems. No other postoperative complications have been observed. 

### 3.2. iMRI

The time from the end of surgery to the start of the iMRI examination was approximately 30 min. During this time, the child was prepared for the iMRI and all necessary arrangements were made for the iMRI to move into the “scanning position”. The total iMRI scan time was about 14 min for the three basic sequences, with a maximum duration of around 25 min with additional sequences. In all patients, the important anatomical structures (urinary bladder, prostate, rectum, urethra, ureters, and intestine) could be visualized in adequate quality. The iMRI allowed precise anatomical localization of the BT tubes and an accurate contouring of the target volume. No patient experienced an adverse event during the scan period.

### 3.3. BT Tubes

A median of eight tubes were placed for BT (range 7–10). In 6 out of 12 patients, BT tubes had to be revised. All tube misplacements were detected by iMRI. The attending radiologist and the radiation oncologist in charge indicated the need for revision. The reasons for this were too large of a distance between two tubes in five cases and a tube placed in the rectal wall in one case. In none of these six patients did a delay in the start of irradiation occur. In the historical group with a total of 20 patients (20 BP-RMS), correction or new placement of BT tubes was necessary in 5 patients. In these cases, the main reason for revision was also a distance between two tubes that was too large (n = 5). In three of these patients, the irradiation start was delayed and could not be started until postoperative day 3.

## 4. Discussion

This study describes our initial experience with an iMRI for BT tube control and irradiation planning in children with BP and perianal RMS. So far, we have successfully applied this concept in 12 children with these diagnoses. 

The difficulty that arises when placing the BT tubes, especially in children with BP-RMS, is the fact that the course of the tubes cannot be seen over a certain distance. In most cases, however, this is exactly the critical area and thereby a large part of the target volume of the irradiation field, in which exact positioning of the tubes is essential. Since we have been using the concept of organ-preserving tumor resection in combination with 3D-planned BT in our department, cross-sectional imaging has been used to control the position of the tubes and to plan the irradiation. Initially, we carried out only a CT scan and in the course of time we added an MRI. The reason for this was the superior tissue resolution of MRI compared to CT. Anatomical structures, especially the organs at risk such as the rectum, urethra, etc., and the position of the BT tubes in relation to the anatomical structures can be better visualized. In addition, the target volume for irradiation can be more precisely defined. The decision of whether a tube revision is necessary can be made with MRI alone. Since January 2021, we have been using iMRI. For technical reasons, we cannot yet dispense with the CT as it is needed for exact radiation dose calculations based on electron densities of different tissues and the exact position of the radioactive sources. An adaptation of the BT planning software and spatial resolution of MRI might make this possible in the future. The main advantages of not using CT would be additional time saving and less radiation exposure for the young patients. Up until now, the two methods have been considered complementary. 

Compared to HDR-BT, the pulsed dose rate (PDR)-BT used in Paris seems to have an advantage in terms of BT tube placement—fewer tubes are used for irradiation and are being placed with more standardization and with less defined distances [11]. The differences in the influence of precise positioning with advantage for PDR might be explained by the lower single dose application per fraction and the estimated repair capacity between the pulses. For PDR, certain structural and personnel requirements are necessary, which are not given everywhere, including at our institution. However, with regard to the oncological and also the functional outcome, there are no relevant differences when using the two different concepts of organ-preserving tumor resection and BT [24,25].

By using iMRI and thus eliminating the need for transportation between the OR unit and the MRI, we were able to save a lot of time and start radiation therapy in all patients at the scheduled time. In the historical cohort, this was not possible in 3 out of 20 patients, mainly because of the use and availability of the MRI outside the OR unit and the fact that potential difficult revisions of BT tubes had to be performed the day after when the experienced surgical team was available again. It cannot be assumed that a delayed start of irradiation by one day leads to a worse oncological outcome. However, the fact that our patients have to be sedated and undergo muscle relaxation from the beginning of the tumor resection until the end of BT in order to prevent BT tube dislocation, prolongs the time under anesthesia and likely increases the associated side effects [26]. 

iMRI cannot prevent misplacement of BT tubes but gives the ability to correct the misplacement within the same procedure. We have investigated different methods to optimize tube placement, like transrectal sonography, and are still evaluating different other modalities. However, we have not yet found a suitable tool that displays the anatomical structures as precisely as an MRI.

In the literature, there are other examples of iMRI application in children, such as in children with imperforate anus [27,28,29], and bladder exstrophy [30]. However, this imaging technique has not become generally accepted for these indications. In our study, iMRI has been proven to be effective in tube control and BT planning in children with BP and perianal RMS. It is now used as a standard for all children with these diagnoses and after the combination treatment of organ-preserving tumor resection and BT. In the future, we are planning to expand the use of iMRI for other indications as well.

This study is the first report of the use of iMRI in the combination therapy of BT and organ-preserving tumor resection in children with BP and perianal RMS and describes the experience of a single center with this method. This study has limitations. The results are based on a small number of cases due to the low incidence of the diseases studied and the even lower number of patients who are suitable for this individualized combination therapy [1,2,9]. The incidence is even less in perianal RMS than in BP-RMS [9]. A separate report on perianal RMS would not be meaningful, so these patients were included in this study. This also seems justified as the focus of the study is on the use of iMRI as a tool to improve the clinical workflow of the combination therapy, which is performed in a similar way in both diseases.

Due to the small number of cases and the low statistical power, the analysis was descriptive only. Patients were prospectively enrolled in the study. However, it was not possible to randomize a significant number of patients, so historical data were used for comparison. This study must therefore be regarded as the first report of the use of a new method for a known application, as a proof of principle.

## 5. Conclusions

In this initial study, by using iMRI, we have been able to increase patient safety and therapy quality by eliminating risky transports under anesthesia between OR unit and the MRI. In addition, delay of radiotherapy start and thus prolonged time under anesthesia can be avoided with this approach in case of necessary surgical revision.

## Figures and Tables

**Table 1:** Patient characteristics and outcome.

Patient	Diagnosis	Age at Local Therapy (Months)	Weight at Local Therapy (kg)	Follow-Up (Months)	Follow-Up Findings
1	BP-RMS	21	10.3	23	NED
2	Perianal RMS	20	11.9	22	NED
3	BP-RMS	28	11.4	7	Dead ^1^
4	BP-RMS	35	10.5	8	Dead ^2^
5	BP-RMS	28	12.0	16	NED
6	BP-RMS	32	12.6	7	NED
7	BP-RMS	24	10.8	13	NED
8	BP-RMS	57	18.5	12	NED
9	BP-RMS	30	12.0	9	NED
10	BP-RMS	13	9.0	6	NED
11	Perianal RMS	22	14.6	0	N/A
12	BP-RMS	73	20.0	0	N/A

BP bladder/prostate; RMS rhabdomyosarcoma; NED no evidence of disease; N/A not available due to follow-up period being too short; ^1^ due to tumor progress; ^2^ non oncological reason.

## Data Availability

The data presented in this study are available in this article.
